# Can a new scoring system reliably predict failed facemask ventilation?

**DOI:** 10.1007/s00540-020-02793-9

**Published:** 2020-05-21

**Authors:** Hans-Joachim Priebe

**Affiliations:** Department of Anesthesiology and Critical Care, Medical Center University of Freiburg, Hugstetter Straße 55, 79095 Freiburg, Germany

**Keywords:** Airway management, Facemask ventilation, Scoring system

To the Editor:

I read with interest the publication by Saito et al. [1]. A couple of clarifications would be welcomed.

The data do not necessarily support the authors’ conclusion that the new scoring system can predict failed facemask ventilation (FMV). The positive predictive values (PPV) of low and high-risk scores for failed FMV are 5.6% and 0.004%, respectively. Such low PPVs can hardly be considered clinically useful in predicting failed FMV. In general, whenever the incidence of an event is very low (like in the case of failed FMV), the PPV will always be low, and the negative predictive value (NPV) high.

In 13 of the 20 cases of failed FMV, a supraglottic airway was successfully inserted. Such 65% success rate seems to contradict the authors’ view that the proposed scoring system is also useful in predicting difficult insertion of, or ventilation through a supraglottic airway.

Specific description of the characteristic of failed FMV and of the clinical management (e.g., expertise of anesthesiologist, head positioning, anesthetic technique and depth, use of neuromuscular blocking drug) of those 20 patients with failed FMV would be very helpful. For example, administration of a neuromuscular blocking drug (NMBD) immediately following induction of anesthesia tends to facilitate FMV [2]. It would thus be clinically relevant to know whether NMBDs had been administered before any attempt at FMV, and before attempt at insertion of a supraglottic airway after FMV had failed.
